# Supplementary figures and images for: The prognostic impact of lymph node dissection for clinically node-negative upper urinary tract urothelial carcinoma in patients who are treated with radical nephroureterectomy
Source: PLoS One. 2022 Dec 1;17(12):e0278038. doi: 10.1371/journal.pone.0278038 (PMC9714942; doi:10.1371/journal.pone.0278038)

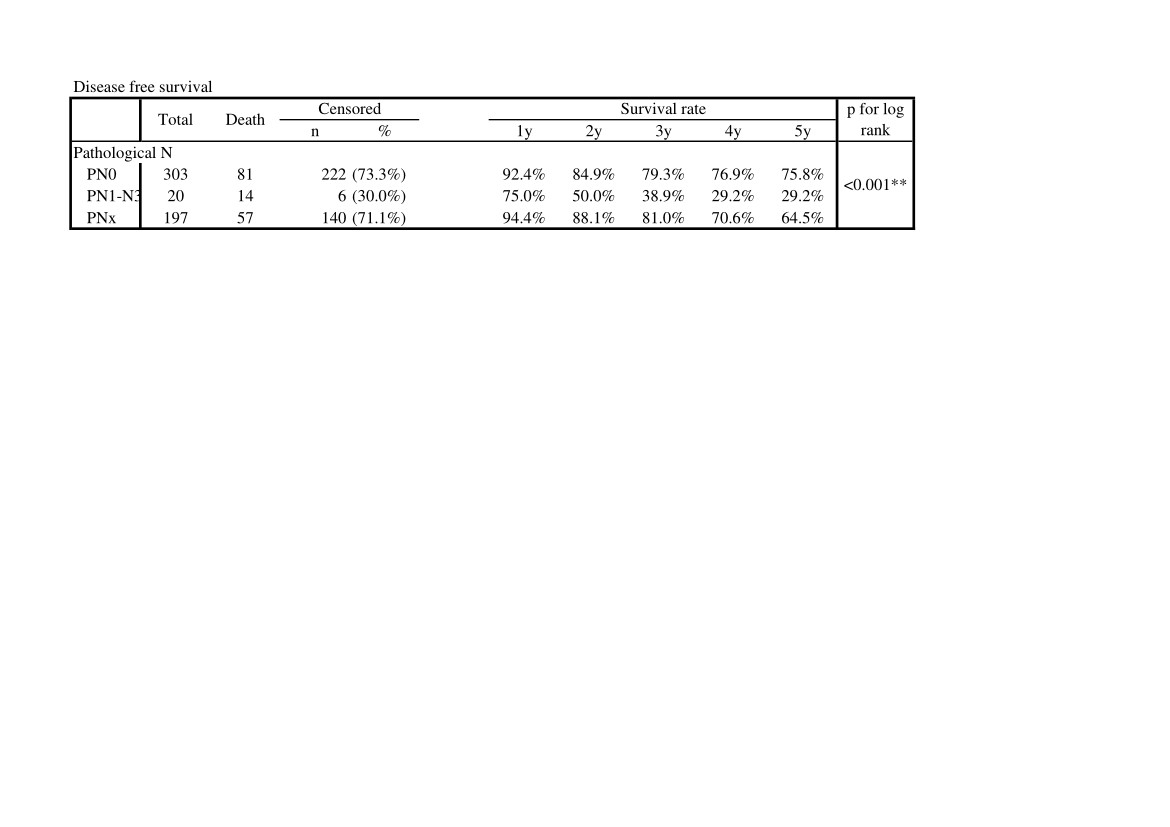

Supplement: S1 Table — Kaplan-Meier analysis of DFS for 520 patients with pathologically proved lymph node status (pN1–3 and pN0) or without LND (pNx) in cN0 UTUC undergoing RNU with BCE. (TIF) [file pone.0278038.s001.tif]

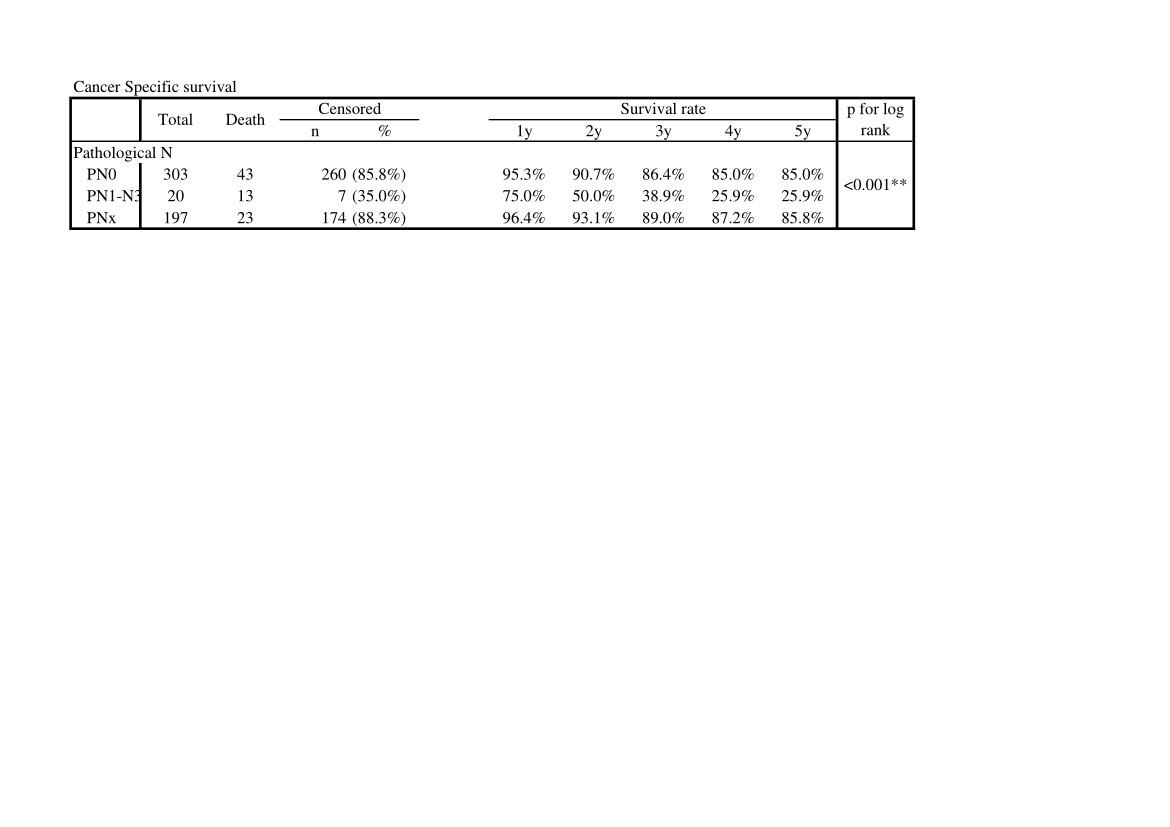

Supplement: S2 Table — Kaplan-Meier analysis of CSS for 520 patients with pathologically proved lymph node status (pN1–3 and pN0) or without LND (pNx) in cN0 UTUC undergoing RNU with BCE. (TIF) [file pone.0278038.s002.tif]

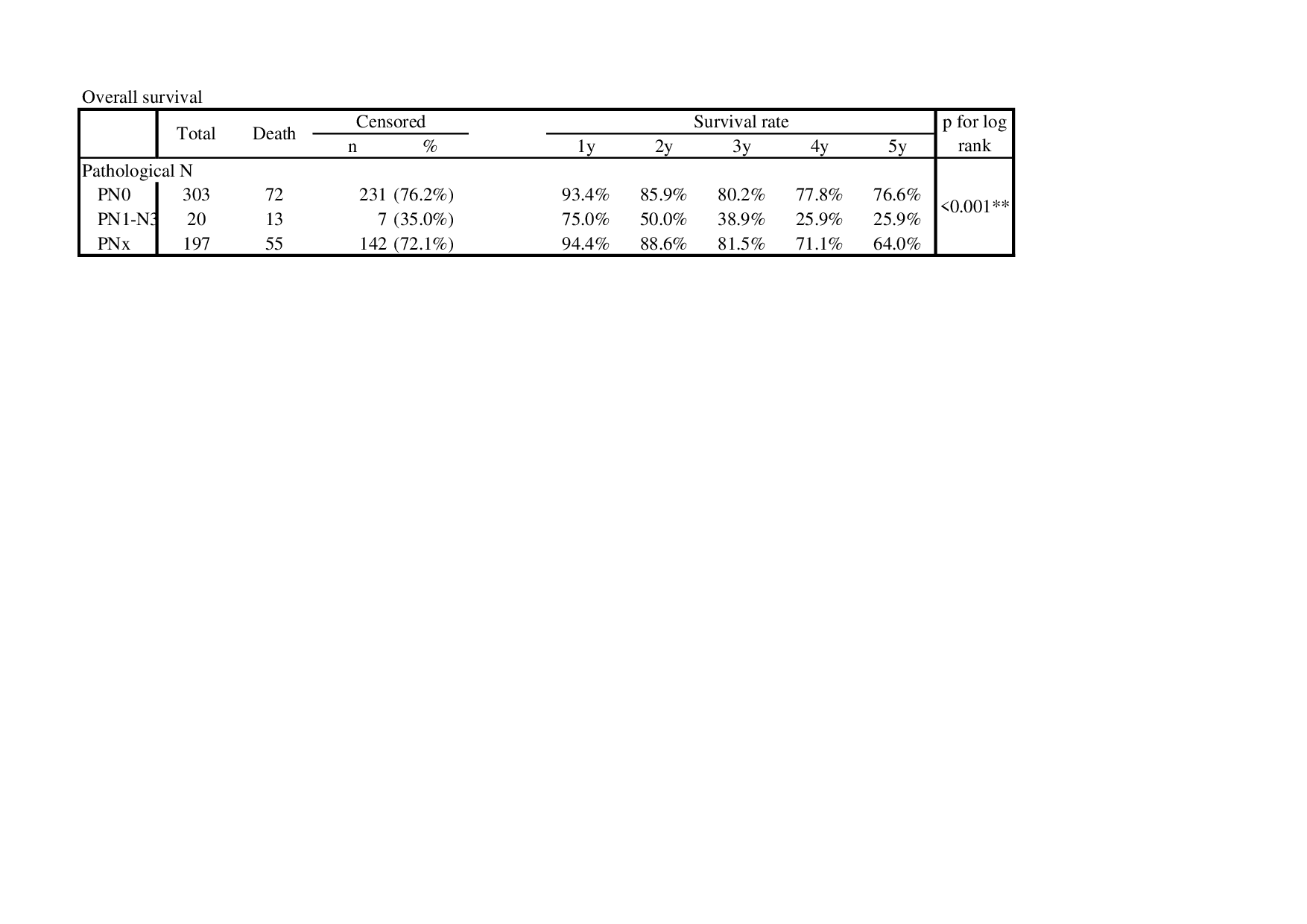

Supplement: S3 Table — Kaplan-Meier analysis of OS for 520 patients with pathologically proved lymph node status (pN1–3 and pN0) or without LND (pNx) in cN0 UTUC undergoing RNU with BCE. (TIF) [file pone.0278038.s003.tif]
