# Supplementary material for: The prognostic impact of lymph node dissection for clinically node-negative upper urinary tract urothelial carcinoma in patients who are treated with radical nephroureterectomy
Source: PLoS One. 2022 Dec 1;17(12):e0278038. doi: 10.1371/journal.pone.0278038 (PMC9714942; doi:10.1371/journal.pone.0278038)
Supplement: Attachment — Submitted filename: Response to Reviewers.docx [file pone.0278038.s004.docx]

Dear Reviewers

Thanks so much for revising my manuscript. The following are individual responses to the reviewers.

To: Reviewer #1

1. Introduction section : we added systemic treatment scenario for UTUC with the recently published papers you suggested (PMID: 34387596; PMID: 33516645 )
2. Discussion section: we summarized and expand our discussion

- The potential of our study may indicate that LND with RNU still had a prediction of a poor prognosis and allow optimal tumor staging for further treatment.
- Further prospective and well‐controlled clinical trials should be done to better establish the impact of lymph node dissection for UTUC in patients with CN0 disease.

3. We added more limitations of this study
4. We had arranged a linguistic revision this time

To: Reviewer #2

1. Title: The title changed and modified to " The prognostic impact of lymph node dissection for clinically node-negative upper urinary tract urothelial carcinoma in patients who are treated with radical nephroureterectomy" as your suggestion.
2. General: We tried to improve the language quality of our revised manuscript this time
3. Abstract: Conclusion: "patients still had a poor prognosis" was mentioned in this section as your suggestion.
4. Materials and Methods:
   - The recorded dissected regions and the extent of LND and adjuvant chemotherapy were mentioned in this section.
5. Result: we had revised this section as your suggestion
   - We used mean follow-up duration with months and SD.
   - Adjuvant chemotherapy results, number of causes of mortality, the estimated OS at 2 and 5 years, and disease recurrence were mentioned in results or tables.
6. Tables:
   - All abbreviations were mentioned below the tables.
   - Table 2 was revised, and included CSS, OS, and DFS (the data also corrected)
7. Discussion:
   - We summarized and expand our discussion as your suggestion.
   - The CSS, OS, and DFS were discussed.
   - Add more limitations of our study.

To: Reviewer #3

1. We had arranged a linguistic revision this time
2. All results were given in the Results section as your suggestion (removed from Materials & Methods section)
3. We provided the data about how many surgeons performed the surgeries and what were their levels of expertise.
4. We used mean follow-up duration with months and SD
5. We made it consistent of lymphovascular invasion (LVI) as your suggestion.
6. Table 2 was revised, and included CSS, OS and DFS.
7. We added POUT trial in the Discussion section as your suggestion about the use of NAC/AC in UTUC management.
8. We started directly with our findings and then compared them with that of previously published papers in discussion section as your suggestion.
9. “LND should be considered when the characteristics of high-grade tumor and LVI are detected in the patients. Do the authors mean that we should take these parameters into account from the pathology report of URS+biopsy? During RNU, we cannot see the tumor and we cannot have an impression that it is high grade. Please clarify this.”

🡪 It’s was our mistake and this paragraph should be “for patients with characteristics of high grade tumor, positive lymphovascular invasion or positive surgical margin, additional AC should be considered because of the risk of lymph node metastasis as implicated in our study”, not “LND”. Thanks for your correction.

10. The selection criteria of LND for cN0 patients was mentioned in limitation
    🡪 Due to the different strategy of LND that resulted from clinical circumstances, surgeons’ personal preferences and evolution of surgical techniques, there may have been potential selection biases.
11. “I have not seen the result of 6.2% rate of occult LN metastasis in the Results section, however, this is written in the Conclusion parts of the text and the abstract. Please clarify this.”
    🡪 The 6.2% (20 patients with pN+/ 323 patients who underwent LND) of our patients with clinically proven occult LN metastasis. Sorry about our unclear description.

We look forward to hearing good news about our revised manuscript.

Kind regards,

Hsiang-Chen Hsieh, MD.

Taichung Veteran General Hospital, Taiwan

Department of Surgery, Division of Urology

Phone: +886-975-960069

E-mail: stilllove3q@hotmail.com

Chia-Yen Lin, MD (Corresponding Author)

Taichung Veteran General Hospital, Taiwan

Department of Surgery, Division of Urology

Mail: lcyhank.tw@gmail.com
